# 1-[(1*S*,6*R*,7*S*,9*R*)-8,8-Di­bromo-5,5,9-tri­methyl­tri­cyclo­[4.4.0.1^7,9^]decan-1-yl]ethanone

**DOI:** 10.1107/S1600536814005431

**Published:** 2014-03-15

**Authors:** Mohamed Zaki, Ahmed Benharref, Lahcen El Ammari, Mohamed Saadi, Moha Berraho

**Affiliations:** a Laboratoire de Chimie Biomoléculaires, Substances Naturelles et Réactivité, URAC16, Faculté des Sciences, Semlalia, BP 2390 Bd My Abdellah, 40000 Marrakech, Morocco; b Laboratoire de Chimie du Solide Appliquée, Faculté des Sciences, Université Mohammed V-Agdal, BP 1014, Avenue Ibn Battouta, Rabat, Morocco

## Abstract

The title compound, C_16_H_24_Br_2_O, was synthesized by three steps from β-himachalene (3,5,5,9-tetra­methyl-2,4a,5,6,7,8-hexa­hydro-1*H*-benzo­cyclo­heptene), which was isolated from essential oil of the Atlas cedar *cedrus atlantica*. The asymmetric unit contains two independent mol­ecules with almost identical conformations. Each mol­ecule is built up from two fused six-membered rings, one having a chair conformation and the other a boat conformation, and an additional three-membered ring arising from the reaction of himachalene with di­bromo­carbene. In the crystal, there are no significant intermolecular interactions present. The absolute structure of the title compound was confirmed by resonance scattering.

## Related literature   

For background to the reactivity and biological properties of β-himachalene, see: El Haib *et al.* (2011 ▶); El Jamili *et al.* (2002 ▶); Daoubi *et al.* (2004 ▶). For a related structure, see: Benharref *et al.* (2013 ▶). For ring conformational analysis, see: Cremer & Pople (1975 ▶).
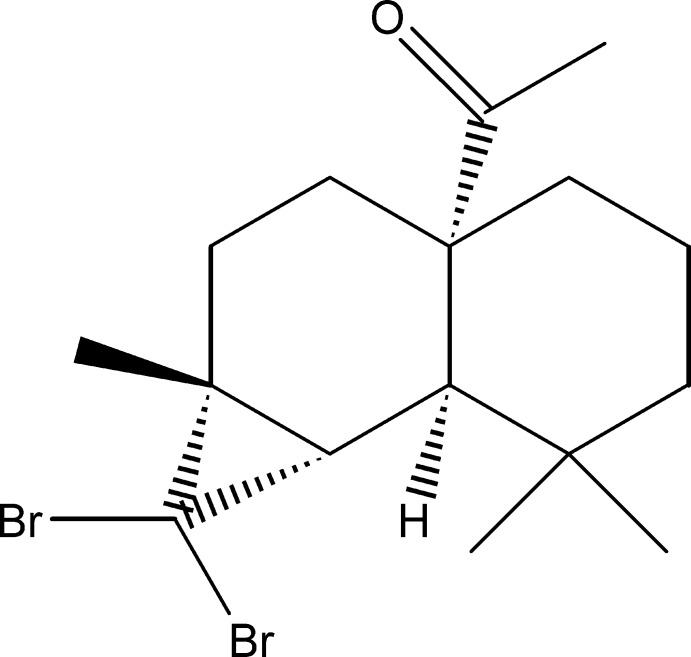


## Experimental   

### 

#### Crystal data  

C_16_H_24_Br_2_O
*M* *_r_* = 392.17
Orthorhombic, 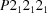

*a* = 13.5013 (19) Å
*b* = 14.042 (2) Å
*c* = 17.213 (3) Å
*V* = 3263.3 (8) Å^3^
*Z* = 8
Mo *K*α radiation
μ = 4.96 mm^−1^
*T* = 298 K
0.5 × 0.03 × 0.03 mm

#### Data collection  

Bruker X8 APEX CCD area-detector diffractometer
Absorption correction: multi-scan (*SADABS*; Bruker, 2009) *T*_min_ = 0.557, *T*_max_ = 0.747
22468 measured reflections
6542 independent reflections
5262 reflections with *I* > 2σ(*I*)
*R*_int_ = 0.040

#### Refinement  

*R*[*F* ^2^ > 2σ(*F* ^2^)] = 0.031
*wR*(*F* ^2^) = 0.060
*S* = 1.02
6540 reflections
351 parameters
H-atom parameters constrained
Δρ_max_ = 0.27 e Å^−3^
Δρ_min_ = −0.39 e Å^−3^
Absolute structure: Flack & Bernardinelli (2000 ▶)
Absolute structure parameter: −0.005 (8)

### 

Data collection: *APEX2* (Bruker, 2009 ▶); cell refinement: *SAINT* (Bruker, 2009 ▶); data reduction: *SAINT*; program(s) used to solve structure: *SHELXS97* (Sheldrick, 2008 ▶); program(s) used to refine structure: *SHELXL97* (Sheldrick, 2008 ▶); molecular graphics: *ORTEP-3 for Windows* (Farrugia, 2012 ▶); software used to prepare material for publication: *WinGX* (Farrugia, 2012 ▶).

## Supplementary Material

Crystal structure: contains datablock(s) I. DOI: 10.1107/S1600536814005431/bt6967sup1.cif

Structure factors: contains datablock(s) I. DOI: 10.1107/S1600536814005431/bt6967Isup2.hkl

CCDC reference: 990889

Additional supporting information:  crystallographic information; 3D view; checkCIF report

## Figures and Tables

**Fig. 1.**
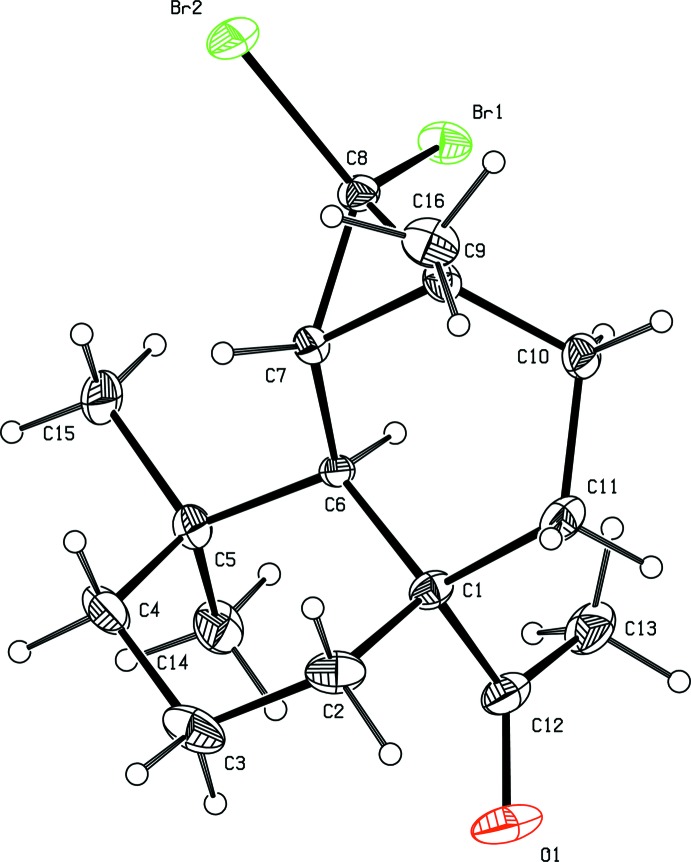
: Perspective view of one of the two molecules in the asymmetric unit of the title compound with the atom-labelling scheme. Displacement ellipsoids are drawn at the 30% probability. level. H atoms are represented as small spheres of arbitrary radii.

## supplementary crystallographic information

### 1. Comment

The bicyclic sesquiterpene β-himachalene is the main constituent of the essential oil of the Atlas cedar (Cedrus atlantica) (El Haib *et al.*, 2011). The reactivity of this sesquiterpene and its derivatives has been studied extensively by our team in order to prepare new products having biological properties (El Jamili *et al.*, 2002; Benharref *et al.*, 2013). Indeed, these compounds were tested, using the food poisoning technique, for their potential antifungal activity against phytopathogen Botrytis cinerea (Daoubi *et al.*, 2004). In this paper we present the crystal structure of the title compound, (1*S*, 6*R*,7*S*, 9*R*)-1-acetyl-8,8-dibromo-5,5,9- trimethyltricyclo[4.4.0,1^7,9^]decane. The asymmetric unit of the title compound contains two independent molecules of similar geometry (Fig. 1). Each molecule contains two fused six-membered rings, which are fused to a three-membered ring as shown in Fig.1. In both molecules, one of the six-membered ring has a chair conformation as indicated by the total puckering amplitude QT = 0.543 (4) Å and spherical polar angle θ_2_ = 177.7 (4)° with φ_2_ = 1(11)°, whereas the other six-membered ring displays a boat conformation with QT = 0.725 (3) Å, θ_2_ = 92.2 (2)° (Cremer & Pople, 1975). Owing to the presence of Br atoms, the absolute configuration could be fully confirmed, by refining the Flack parameter as C1(S), C6(*R*), C7(S) and C9(*R*).

### 2. Experimental

To obtain the title compound, BF_3_–Et_2_O (1 ml) was added dropwise to a solution of (1*S*,2*R*,7*R*,8S,10*R*)-9,9-dibromo- 1α, 2α- epoxy -2, 6, 6, 10-tetramethyltricyclo [5.5.0.0^8^,^10^] dodecane (1 g, 2.5 mmol) in 60 ml of dichloromethane at 195 K under nitrogen. The reaction mixture was stirred for two hours at a constant temperature of 195 K and the left at ambient temperature for 24 h. Water (60 ml) was added in order to separate the two phases, and the organic phase was dried and concentrated. The residue obtained was chromatographed on silica-gel eluting with hexane-ethyle acetate (98/2), which allowed the isolation of pure(1*S*, 6*R*,7*S*, 9*R*)-12-acetyl- 8,8-dibromo-5,5,9-trimethyltricyclo[4.4.0,1^7^,^9^]decane in a yield of 70% (686 mg, 1.75 mmol). The title compound was recrystallized from its cyclohexane solution.

### 3. Refinement

All H atoms were fixed geometrically and treated as riding with C—H = 0.96 Å (methyl), 0.97 Å (methylene), 0.98 Å (methine) with *U*_iso_(H) = 1.2*U*_eq_ (methylene, methine) or *U*_iso_(H) = 1.5*U*_eq_ (methyl).

### Crystal data

**Table:**

C_16_H_24_Br_2_O	*D*_x_ = 1.596 Mg m^−^^3^
*M**_r_* = 392.17	Mo *K*α radiation, λ = 0.71073 Å
Orthorhombic, *P*2_1_2_1_2_1_	Cell parameters from 6542 reflections
*a* = 13.5013 (19) Å	θ = 2.4–26.4°
*b* = 14.042 (2) Å	µ = 4.96 mm^−^^1^
*c* = 17.213 (3) Å	*T* = 298 K
*V* = 3263.3 (8) Å^3^	Block, colourless
*Z* = 8	0.5 × 0.03 × 0.03 mm
*F*(000) = 1584	

### Data collection

**Table:**

Bruker X8 APEX CCD area-detector diffractometer	6542 independent reflections
Radiation source: fine-focus sealed tube	5262 reflections with *I* > 2σ(*I*)
Graphite monochromator	*R*_int_ = 0.040
φ and ω scans	θ_max_ = 26.4°, θ_min_ = 2.4°
Absorption correction: multi-scan (*SADABS*; Bruker, 2009)	*h* = −15→16
*T*_min_ = 0.557, *T*_max_ = 0.747	*k* = −12→17
22468 measured reflections	*l* = −17→21

### Refinement

**Table:**

Refinement on *F*^2^	Secondary atom site location: difference Fourier map
Least-squares matrix: full	Hydrogen site location: inferred from neighbouring sites
*R*[*F*^2^ > 2σ(*F*^2^)] = 0.031	H-atom parameters constrained
*wR*(*F*^2^) = 0.060	*w* = 1/[σ^2^(*F*_o_^2^) + (0.0104*P*)^2^] where *P* = (*F*_o_^2^ + 2*F*_c_^2^)/3
*S* = 1.02	(Δ/σ)_max_ = 0.001
6540 reflections	Δρ_max_ = 0.27 e Å^−^^3^
351 parameters	Δρ_min_ = −0.39 e Å^−^^3^
0 restraints	Absolute structure: Flack & Bernardinelli (2000)
Primary atom site location: structure-invariant direct methods	Absolute structure parameter: −0.005 (8)

### Special details

**Table:**

Geometry. All e.s.d.'s (except the e.s.d. in the dihedral angle between two l.s. planes) are estimated using the full covariance matrix. The cell e.s.d.'s are taken into account individually in the estimation of e.s.d.'s in distances, angles and torsion angles; correlations between e.s.d.'s in cell parameters are only used when they are defined by crystal symmetry. An approximate (isotropic) treatment of cell e.s.d.'s is used for estimating e.s.d.'s involving l.s. planes.
Refinement. Refinement of *F*^2^ against all reflections. The weighted *R*-factor *wR* and goodness of fit *S* are based on *F*^2^, conventional *R*-factors *R* are based on *F*, with *F* set to zero for negative *F*^2^. The threshold expression of *F*^2^ > 2σ(*F*^2^) is used only for calculating *R*-factors(gt) *etc*. and is not relevant to the choice of reflections for refinement. *R*-factors based on *F*^2^ are statistically about twice as large as those based on *F*, and *R*- factors based on all data will be even larger.

### Fractional atomic coordinates and isotropic or equivalent isotropic displacement parameters (Å^2^)

**Table:**

	*x*	*y*	*z*	*U*_iso_*/*U*_eq_	
C1	0.7516 (2)	1.0732 (2)	−0.03701 (18)	0.0342 (8)	
C2	0.6483 (3)	1.1166 (3)	−0.0456 (2)	0.0518 (10)	
H2A	0.6508	1.1658	−0.0851	0.062*	
H2B	0.6032	1.0676	−0.0636	0.062*	
C3	0.6068 (3)	1.1596 (3)	0.0284 (2)	0.0628 (12)	
H3A	0.6465	1.2142	0.0431	0.075*	
H3B	0.5398	1.1815	0.0189	0.075*	
C4	0.6060 (3)	1.0889 (3)	0.0941 (2)	0.0544 (10)	
H4A	0.5600	1.0379	0.0819	0.065*	
H4B	0.5827	1.1202	0.1409	0.065*	
C5	0.7090 (2)	1.0460 (3)	0.10960 (19)	0.0394 (9)	
C6	0.7513 (2)	1.0035 (2)	0.03386 (15)	0.0250 (7)	
H6	0.8204	0.9864	0.0442	0.030*	
C7	0.6976 (2)	0.9125 (2)	0.01156 (17)	0.0298 (8)	
H7	0.6264	0.9124	0.0228	0.036*	
C8	0.7455 (2)	0.8170 (2)	0.01354 (18)	0.0329 (8)	
C9	0.7266 (2)	0.8636 (2)	−0.06406 (17)	0.0320 (8)	
C10	0.8094 (2)	0.9159 (2)	−0.1044 (2)	0.0393 (9)	
H10A	0.8216	0.8877	−0.1549	0.047*	
H10B	0.8696	0.9114	−0.0739	0.047*	
C11	0.7797 (3)	1.0195 (3)	−0.11368 (18)	0.0435 (9)	
H11A	0.7235	1.0225	−0.1488	0.052*	
H11B	0.8340	1.0532	−0.1382	0.052*	
C12	0.8323 (3)	1.1502 (3)	−0.0304 (2)	0.0452 (9)	
C13	0.9368 (3)	1.1193 (3)	−0.0122 (2)	0.0568 (11)	
H13A	0.9824	1.1549	−0.0437	0.085*	
H13B	0.9439	1.0526	−0.0231	0.085*	
H13C	0.9506	1.1309	0.0417	0.085*	
C14	0.7777 (3)	1.1229 (3)	0.1434 (2)	0.0547 (11)	
H14A	0.7492	1.1479	0.1902	0.082*	
H14B	0.7855	1.1734	0.1062	0.082*	
H14C	0.8412	1.0955	0.1547	0.082*	
C15	0.6990 (3)	0.9672 (3)	0.1702 (2)	0.0553 (11)	
H15A	0.6551	0.9188	0.1512	0.083*	
H15B	0.6728	0.9935	0.2175	0.083*	
H15C	0.7629	0.9398	0.1802	0.083*	
C16	0.6480 (3)	0.8239 (3)	−0.1165 (2)	0.0517 (10)	
H16A	0.6738	0.7697	−0.1438	0.077*	
H16B	0.6282	0.8717	−0.1532	0.077*	
H16C	0.5918	0.8049	−0.0861	0.077*	
Br1	0.87872 (2)	0.80148 (3)	0.05270 (2)	0.04524 (10)	
Br2	0.66521 (3)	0.71109 (3)	0.04601 (3)	0.05635 (12)	
O1	0.8142 (2)	1.23284 (19)	−0.04537 (18)	0.0746 (9)	
C17	0.8403 (2)	0.5321 (2)	0.30776 (18)	0.0353 (8)	
C18	0.9200 (3)	0.4590 (3)	0.3290 (2)	0.0536 (11)	
H18A	0.9638	0.4872	0.3674	0.064*	
H18B	0.8881	0.4046	0.3531	0.064*	
C19	0.9822 (3)	0.4239 (3)	0.2615 (2)	0.0645 (12)	
H19A	1.0214	0.4762	0.2412	0.077*	
H19B	1.0274	0.3750	0.2796	0.077*	
C20	0.9176 (3)	0.3837 (3)	0.1974 (2)	0.0532 (10)	
H20A	0.8836	0.3276	0.2166	0.064*	
H20B	0.9593	0.3643	0.1543	0.064*	
C21	0.8409 (2)	0.4558 (2)	0.16839 (19)	0.0358 (8)	
C22	0.7784 (2)	0.4940 (2)	0.23762 (17)	0.0297 (7)	
H22	0.7397	0.5479	0.2178	0.036*	
C23	0.7047 (2)	0.4204 (2)	0.26590 (18)	0.0319 (8)	
H23	0.7282	0.3544	0.2648	0.038*	
C24	0.5956 (2)	0.4328 (2)	0.25512 (19)	0.0365 (8)	
C25	0.6391 (2)	0.4460 (2)	0.33457 (19)	0.0331 (8)	
C26	0.6622 (2)	0.5442 (3)	0.3653 (2)	0.0411 (9)	
H26A	0.6258	0.5554	0.4130	0.049*	
H26B	0.6426	0.5920	0.3276	0.049*	
C27	0.7733 (2)	0.5508 (3)	0.38072 (18)	0.0437 (9)	
H27A	0.7906	0.5052	0.4208	0.052*	
H27B	0.7880	0.6139	0.4007	0.052*	
C28	0.8851 (3)	0.6296 (3)	0.2910 (2)	0.0501 (10)	
C29	0.8180 (3)	0.7092 (3)	0.2652 (2)	0.0614 (11)	
H29A	0.8507	0.7691	0.2729	0.092*	
H29B	0.7580	0.7077	0.2951	0.092*	
H29C	0.8024	0.7014	0.2112	0.092*	
C30	0.8932 (3)	0.5378 (3)	0.1257 (2)	0.0517 (10)	
H30A	0.9371	0.5699	0.1609	0.078*	
H30B	0.8446	0.5820	0.1067	0.078*	
H30C	0.9305	0.5128	0.0828	0.078*	
C31	0.7714 (3)	0.4076 (3)	0.1084 (2)	0.0541 (11)	
H31A	0.8092	0.3880	0.0640	0.081*	
H31B	0.7213	0.4521	0.0925	0.081*	
H31C	0.7406	0.3530	0.1317	0.081*	
C32	0.6193 (3)	0.3715 (3)	0.3973 (2)	0.0520 (10)	
H32A	0.5559	0.3833	0.4206	0.078*	
H32B	0.6699	0.3751	0.4364	0.078*	
H32C	0.6196	0.3091	0.3744	0.078*	
Br3	0.54217 (3)	0.53917 (3)	0.19802 (2)	0.04941 (11)	
Br4	0.51892 (3)	0.32022 (3)	0.23283 (2)	0.06195 (13)	
O2	0.9721 (2)	0.6461 (2)	0.30335 (19)	0.0872 (10)	

### Atomic displacement parameters (Å^2^)

**Table:**

	*U*^11^	*U*^22^	*U*^33^	*U*^12^	*U*^13^	*U*^23^
C1	0.0425 (19)	0.0318 (19)	0.0284 (19)	−0.0034 (16)	−0.0046 (16)	0.0053 (15)
C2	0.060 (2)	0.041 (2)	0.054 (2)	0.0080 (18)	−0.018 (2)	0.009 (2)
C3	0.048 (2)	0.052 (3)	0.089 (3)	0.025 (2)	−0.011 (2)	−0.007 (2)
C4	0.051 (2)	0.049 (3)	0.063 (3)	0.0153 (19)	0.011 (2)	−0.014 (2)
C5	0.044 (2)	0.040 (2)	0.0341 (19)	0.0017 (17)	0.0072 (16)	−0.0049 (18)
C6	0.0288 (15)	0.0211 (16)	0.0249 (17)	0.0002 (13)	0.0003 (13)	0.0007 (13)
C7	0.0280 (17)	0.030 (2)	0.0310 (18)	0.0021 (14)	0.0019 (14)	−0.0011 (14)
C8	0.0285 (16)	0.0267 (19)	0.0435 (19)	−0.0038 (15)	−0.0005 (15)	0.0008 (15)
C9	0.0354 (18)	0.0330 (19)	0.0277 (18)	−0.0013 (15)	0.0005 (15)	−0.0085 (15)
C10	0.047 (2)	0.041 (2)	0.0297 (19)	−0.0038 (16)	0.0077 (16)	−0.0060 (16)
C11	0.056 (2)	0.043 (2)	0.0315 (19)	−0.0121 (18)	0.0011 (17)	0.0059 (17)
C12	0.069 (3)	0.034 (2)	0.033 (2)	−0.0144 (19)	0.0010 (19)	0.0014 (17)
C13	0.054 (2)	0.052 (3)	0.064 (3)	−0.024 (2)	0.006 (2)	0.001 (2)
C14	0.067 (3)	0.055 (3)	0.042 (2)	−0.004 (2)	0.003 (2)	−0.0179 (19)
C15	0.077 (3)	0.060 (3)	0.0290 (19)	−0.002 (2)	0.0176 (19)	−0.001 (2)
C16	0.054 (2)	0.047 (2)	0.054 (2)	−0.0055 (19)	−0.0146 (19)	−0.0152 (19)
Br1	0.04248 (19)	0.0378 (2)	0.0554 (2)	0.00669 (16)	−0.01088 (17)	0.00135 (19)
Br2	0.0576 (2)	0.0342 (2)	0.0772 (3)	−0.01247 (18)	0.0117 (2)	0.0086 (2)
O1	0.108 (2)	0.0304 (17)	0.086 (2)	−0.0148 (15)	−0.0083 (19)	0.0137 (15)
C17	0.0281 (16)	0.042 (2)	0.0356 (18)	−0.0008 (16)	−0.0081 (15)	0.0005 (17)
C18	0.045 (2)	0.069 (3)	0.047 (2)	0.005 (2)	−0.0138 (18)	0.007 (2)
C19	0.041 (2)	0.077 (3)	0.075 (3)	0.023 (2)	−0.003 (2)	0.012 (2)
C20	0.052 (2)	0.046 (2)	0.061 (2)	0.0181 (19)	0.015 (2)	0.002 (2)
C21	0.0389 (18)	0.031 (2)	0.0372 (18)	0.0057 (16)	0.0062 (16)	−0.0015 (16)
C22	0.0327 (17)	0.0252 (18)	0.0312 (17)	0.0045 (14)	0.0006 (14)	0.0011 (14)
C23	0.0324 (17)	0.0258 (19)	0.0374 (19)	0.0012 (14)	−0.0019 (15)	0.0062 (15)
C24	0.0353 (18)	0.0303 (19)	0.044 (2)	−0.0014 (15)	−0.0059 (16)	0.0011 (16)
C25	0.0297 (17)	0.034 (2)	0.0351 (18)	0.0026 (15)	0.0022 (14)	0.0030 (16)
C26	0.045 (2)	0.045 (2)	0.0334 (19)	0.0048 (18)	0.0052 (16)	−0.0004 (17)
C27	0.052 (2)	0.045 (2)	0.034 (2)	−0.0074 (18)	−0.0054 (17)	0.0005 (17)
C28	0.049 (2)	0.054 (3)	0.047 (2)	−0.018 (2)	−0.0040 (19)	−0.0065 (19)
C29	0.086 (3)	0.036 (2)	0.062 (2)	−0.009 (2)	−0.003 (2)	0.004 (2)
C30	0.051 (2)	0.053 (3)	0.051 (2)	0.000 (2)	0.0137 (19)	0.012 (2)
C31	0.061 (2)	0.062 (3)	0.039 (2)	0.001 (2)	0.006 (2)	−0.015 (2)
C32	0.055 (2)	0.054 (2)	0.047 (2)	0.006 (2)	0.009 (2)	0.018 (2)
Br3	0.03866 (19)	0.0577 (3)	0.0519 (2)	0.00694 (18)	−0.00959 (18)	0.01018 (19)
Br4	0.0534 (2)	0.0531 (3)	0.0794 (3)	−0.0209 (2)	−0.0020 (2)	−0.0105 (2)
O2	0.0579 (18)	0.085 (2)	0.119 (3)	−0.0333 (18)	−0.020 (2)	0.004 (2)

### Geometric parameters (Å, º)

**Table:**

C1—C2	1.529 (5)	C17—C28	1.524 (5)
C1—C12	1.540 (5)	C17—C18	1.532 (5)
C1—C6	1.564 (4)	C17—C22	1.563 (4)
C1—C11	1.567 (4)	C17—C27	1.570 (5)
C2—C3	1.516 (5)	C18—C19	1.516 (5)
C2—H2A	0.9700	C18—H18A	0.9700
C2—H2B	0.9700	C18—H18B	0.9700
C3—C4	1.506 (5)	C19—C20	1.515 (5)
C3—H3A	0.9700	C19—H19A	0.9700
C3—H3B	0.9700	C19—H19B	0.9700
C4—C5	1.539 (5)	C20—C21	1.531 (4)
C4—H4A	0.9700	C20—H20A	0.9700
C4—H4B	0.9700	C20—H20B	0.9700
C5—C15	1.527 (5)	C21—C30	1.538 (4)
C5—C14	1.537 (5)	C21—C31	1.550 (5)
C5—C6	1.543 (4)	C21—C22	1.556 (4)
C6—C7	1.519 (4)	C22—C23	1.515 (4)
C6—H6	0.9800	C22—H22	0.9800
C7—C8	1.489 (4)	C23—C24	1.496 (4)
C7—C9	1.523 (4)	C23—C25	1.520 (4)
C7—H7	0.9800	C23—H23	0.9800
C8—C9	1.510 (4)	C24—C25	1.500 (4)
C8—Br2	1.924 (3)	C24—Br4	1.928 (3)
C8—Br1	1.933 (3)	C24—Br3	1.928 (3)
C9—C16	1.501 (4)	C25—C26	1.509 (5)
C9—C10	1.506 (4)	C25—C32	1.528 (4)
C10—C11	1.517 (5)	C26—C27	1.526 (5)
C10—H10A	0.9700	C26—H26A	0.9700
C10—H10B	0.9700	C26—H26B	0.9700
C11—H11A	0.9700	C27—H27A	0.9700
C11—H11B	0.9700	C27—H27B	0.9700
C12—O1	1.213 (4)	C28—O2	1.215 (4)
C12—C13	1.509 (5)	C28—C29	1.506 (5)
C13—H13A	0.9600	C29—H29A	0.9600
C13—H13B	0.9600	C29—H29B	0.9600
C13—H13C	0.9600	C29—H29C	0.9600
C14—H14A	0.9600	C30—H30A	0.9600
C14—H14B	0.9600	C30—H30B	0.9600
C14—H14C	0.9600	C30—H30C	0.9600
C15—H15A	0.9600	C31—H31A	0.9600
C15—H15B	0.9600	C31—H31B	0.9600
C15—H15C	0.9600	C31—H31C	0.9600
C16—H16A	0.9600	C32—H32A	0.9600
C16—H16B	0.9600	C32—H32B	0.9600
C16—H16C	0.9600	C32—H32C	0.9600
			
C2—C1—C12	111.9 (3)	C28—C17—C18	111.5 (3)
C2—C1—C6	108.8 (3)	C28—C17—C22	112.0 (3)
C12—C1—C6	112.5 (3)	C18—C17—C22	109.3 (3)
C2—C1—C11	109.4 (3)	C28—C17—C27	103.3 (3)
C12—C1—C11	103.3 (3)	C18—C17—C27	109.0 (3)
C6—C1—C11	110.9 (3)	C22—C17—C27	111.6 (2)
C3—C2—C1	114.5 (3)	C19—C18—C17	115.1 (3)
C3—C2—H2A	108.6	C19—C18—H18A	108.5
C1—C2—H2A	108.6	C17—C18—H18A	108.5
C3—C2—H2B	108.6	C19—C18—H18B	108.5
C1—C2—H2B	108.6	C17—C18—H18B	108.5
H2A—C2—H2B	107.6	H18A—C18—H18B	107.5
C4—C3—C2	111.8 (3)	C20—C19—C18	111.1 (3)
C4—C3—H3A	109.3	C20—C19—H19A	109.4
C2—C3—H3A	109.3	C18—C19—H19A	109.4
C4—C3—H3B	109.3	C20—C19—H19B	109.4
C2—C3—H3B	109.3	C18—C19—H19B	109.4
H3A—C3—H3B	107.9	H19A—C19—H19B	108.0
C3—C4—C5	112.4 (3)	C19—C20—C21	112.4 (3)
C3—C4—H4A	109.1	C19—C20—H20A	109.1
C5—C4—H4A	109.1	C21—C20—H20A	109.1
C3—C4—H4B	109.1	C19—C20—H20B	109.1
C5—C4—H4B	109.1	C21—C20—H20B	109.1
H4A—C4—H4B	107.8	H20A—C20—H20B	107.9
C15—C5—C14	107.7 (3)	C20—C21—C30	109.9 (3)
C15—C5—C4	108.8 (3)	C20—C21—C31	109.8 (3)
C14—C5—C4	109.6 (3)	C30—C21—C31	106.6 (3)
C15—C5—C6	109.3 (3)	C20—C21—C22	110.2 (3)
C14—C5—C6	111.6 (3)	C30—C21—C22	110.9 (3)
C4—C5—C6	109.8 (3)	C31—C21—C22	109.4 (3)
C7—C6—C5	111.2 (3)	C23—C22—C21	111.5 (3)
C7—C6—C1	109.3 (2)	C23—C22—C17	109.7 (2)
C5—C6—C1	114.7 (3)	C21—C22—C17	114.8 (2)
C7—C6—H6	107.1	C23—C22—H22	106.8
C5—C6—H6	107.1	C21—C22—H22	106.8
C1—C6—H6	107.1	C17—C22—H22	106.8
C8—C7—C6	123.0 (2)	C24—C23—C22	121.8 (3)
C8—C7—C9	60.1 (2)	C24—C23—C25	59.7 (2)
C6—C7—C9	118.2 (3)	C22—C23—C25	118.1 (3)
C8—C7—H7	114.8	C24—C23—H23	115.3
C6—C7—H7	114.8	C22—C23—H23	115.3
C9—C7—H7	114.8	C25—C23—H23	115.3
C7—C8—C9	61.0 (2)	C23—C24—C25	61.0 (2)
C7—C8—Br2	117.2 (2)	C23—C24—Br4	117.3 (2)
C9—C8—Br2	119.8 (2)	C25—C24—Br4	119.5 (2)
C7—C8—Br1	120.9 (2)	C23—C24—Br3	121.5 (2)
C9—C8—Br1	121.0 (2)	C25—C24—Br3	121.0 (2)
Br2—C8—Br1	109.63 (15)	Br4—C24—Br3	109.45 (15)
C16—C9—C10	115.4 (3)	C24—C25—C26	120.9 (3)
C16—C9—C8	119.4 (3)	C24—C25—C23	59.4 (2)
C10—C9—C8	119.5 (3)	C26—C25—C23	111.6 (3)
C16—C9—C7	120.0 (3)	C24—C25—C32	119.4 (3)
C10—C9—C7	111.4 (3)	C26—C25—C32	114.5 (3)
C8—C9—C7	58.8 (2)	C23—C25—C32	119.3 (3)
C9—C10—C11	108.6 (3)	C25—C26—C27	108.7 (3)
C9—C10—H10A	110.0	C25—C26—H26A	110.0
C11—C10—H10A	110.0	C27—C26—H26A	110.0
C9—C10—H10B	110.0	C25—C26—H26B	110.0
C11—C10—H10B	110.0	C27—C26—H26B	110.0
H10A—C10—H10B	108.3	H26A—C26—H26B	108.3
C10—C11—C1	115.9 (3)	C26—C27—C17	114.6 (3)
C10—C11—H11A	108.3	C26—C27—H27A	108.6
C1—C11—H11A	108.3	C17—C27—H27A	108.6
C10—C11—H11B	108.3	C26—C27—H27B	108.6
C1—C11—H11B	108.3	C17—C27—H27B	108.6
H11A—C11—H11B	107.4	H27A—C27—H27B	107.6
O1—C12—C13	120.5 (4)	O2—C28—C29	119.4 (4)
O1—C12—C1	120.9 (4)	O2—C28—C17	121.5 (4)
C13—C12—C1	118.4 (3)	C29—C28—C17	118.9 (3)
C12—C13—H13A	109.5	C28—C29—H29A	109.5
C12—C13—H13B	109.5	C28—C29—H29B	109.5
H13A—C13—H13B	109.5	H29A—C29—H29B	109.5
C12—C13—H13C	109.5	C28—C29—H29C	109.5
H13A—C13—H13C	109.5	H29A—C29—H29C	109.5
H13B—C13—H13C	109.5	H29B—C29—H29C	109.5
C5—C14—H14A	109.5	C21—C30—H30A	109.5
C5—C14—H14B	109.5	C21—C30—H30B	109.5
H14A—C14—H14B	109.5	H30A—C30—H30B	109.5
C5—C14—H14C	109.5	C21—C30—H30C	109.5
H14A—C14—H14C	109.5	H30A—C30—H30C	109.5
H14B—C14—H14C	109.5	H30B—C30—H30C	109.5
C5—C15—H15A	109.5	C21—C31—H31A	109.5
C5—C15—H15B	109.5	C21—C31—H31B	109.5
H15A—C15—H15B	109.5	H31A—C31—H31B	109.5
C5—C15—H15C	109.5	C21—C31—H31C	109.5
H15A—C15—H15C	109.5	H31A—C31—H31C	109.5
H15B—C15—H15C	109.5	H31B—C31—H31C	109.5
C9—C16—H16A	109.5	C25—C32—H32A	109.5
C9—C16—H16B	109.5	C25—C32—H32B	109.5
H16A—C16—H16B	109.5	H32A—C32—H32B	109.5
C9—C16—H16C	109.5	C25—C32—H32C	109.5
H16A—C16—H16C	109.5	H32A—C32—H32C	109.5
H16B—C16—H16C	109.5	H32B—C32—H32C	109.5
